# An Unnecessary Russell’s Viper Bite on the Tongue Due to Live Snake Worship and Dangerous First Aid Emphasise the Urgent Need for Stringent Policies

**DOI:** 10.3390/toxins14120817

**Published:** 2022-11-22

**Authors:** Subramanian Senthilkumaran, S. V. Arathisenthil, Jarred Williams, Harry F. Williams, Ponniah Thirumalaikolundusubramanian, Ketan Patel, Sakthivel Vaiyapuri

**Affiliations:** 1 Manian Medical Centre, Erode 638001, Tamil Nadu, India; 2 School of Pharmacy, University of Reading, Reading RG6 6UB, UK; 3 Toxiven Biotech Private Limited, Coimbatore 641042, Tamil Nadu, India; 4 Department of General Medicine, The Tamil Nadu Dr M.G.R Medical University, Chennai 600032, Tamil Nadu, India; 5 School of Biological Sciences, University of Reading, Reading RG6 6UB, UK

**Keywords:** Russell’s viper, live snake worship, snakebite envenomation, snakebite on tongue, airway obstruction, inappropriate first aid, snakebite public awareness

## Abstract

India suffers the highest incidence of snakebite envenomation (SBE) in the world. Rural communities within India and other countries have long-held cultural beliefs surrounding snakes and SBE treatments, with snake statues present in numerous Hindu temples. While most cultural beliefs are well respected and do not affect anyone, some people worship live venomous snakes without any safety precautions. Moreover, they practice various inappropriate first aid and traditional treatments that exacerbate SBE-induced complications. We report an unusual case of SBE on the tongue of a patient who was bitten while worshipping Russell’s viper following the advice of an astrologer based on the appearance of a snake in the patient’s dream. Following the bite, the tongue was deeply incised by the priest as a first aid to mitigate SBE-induced complications. The patient suffered profuse bleeding and swelling of the tongue resulting in difficulties in intubating them. The patient regained consciousness after antivenom administration, intranasal ventilation, and blood removal from the mouth. The tongue underwent extensive surgery to restore movement and function. This report advises caution to those undertaking the extremely risky practice of worshipping live snakes and emphasises the urgent need to develop and enforce policies to mitigate such actions and educate rural communities.

## 1. Introduction

Snakebite envenomation (SBE) is a major healthcare issue which results in around 140,000 deaths and 500,000 permanent disabilities worldwide every year [1,2]. In India alone, there are around 58,000 SBE-induced annual deaths [3]. Snakes have played a central role in many of the ancient traditions and cultures of several religions and tribes across the world [4,5]. Snakes, particularly cobras, are worshipped in many countries including India and it is a common practice to provide milk, eggs, and other foods as offerings to snake statues (or live snakes in some places) [6,7]. While we can fully understand and respect the traditions and cultures of people who take pride and solace in worshipping snake statues, some people practice dangerous, live snake worships [8] without realising the potential consequences on human health, let alone the difficulties in keeping captive snakes.

Rural communities around the world believe in a range of myths and misbeliefs surrounding snakes and SBE treatments. Reluctance to abandon inappropriate traditional misbeliefs acquired over thousands of years can have severe effects on SBE victims. For example, clinicians work hard to tackle SBE complications, but these are often exacerbated by inappropriate first aid and/or treatments [9,10]. Incisions, blood-sucking, application of tourniquets and the burning of bite sites are some of the common practices following SBE [9,11,12]. While most bites normally occur in limbs, some were reported to happen in unusual sites including the tongue of victims [13,14,15,16]. The ‘Big Four’ snakes that include the Russell’s viper (*Daboia russelii*), Indian cobra (*Naja naja*), common krait (*Bungarus caeruleus*) and saw-scaled viper (*Echis carinatus*) are major medically important venomous snake species in India. Specifically, Russell’s viper is responsible for most envenomings, deaths, and morbidities in rural India [9,17]. The venom of Russell’s viper contains a large amount of phospholipase A_2_ (PLA_2_) along with metalloproteases, serine proteases, Kunitz-type inhibitor-like molecules, C-type lectin-like proteins, and other minor protein components [18]. Envenomation from Russell’s viper induces coagulopathies, excessive bleeding, severe pain, swelling, tissue necrosis, acute kidney injury and neurotoxicity [17]. However, the bites from the Indian cobra and common krait predominantly induce neurotoxicity with clinical symptoms such as breathing difficulties, ptosis, blurred vision, and facial paralysis due to a large amount of neurotoxic PLA_2,_ and three-finger toxins present in their venoms [19]. Here, we report an unusual case of Russell’s viper bite to the tongue of a patient while worshipping a live snake specimen in a temple. Following the incident, the temple’s priest severely incised the patient’s tongue. We demonstrate the unusual complications and difficulties in treating this patient and saving their life as well as describe the prospects for the incised tongue.

## 2. Case Report

**Figure 1:**
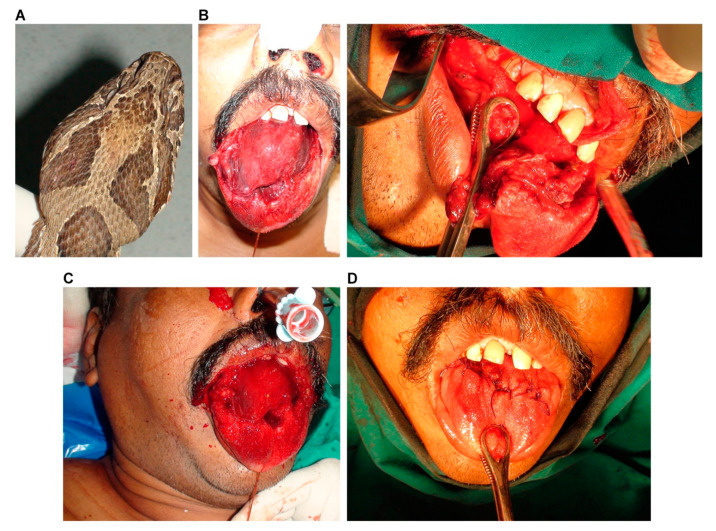
Serious implications of Russell’s viper bite to the tongue of a patient and subsequent deep incision as first aid. (**A**) The offending snake was identified as Russell’s viper by a herpetologist. (**B**) Excessive swelling and laceration of the tongue. (**C**) The successful intubation of the patient via the intranasal route. (**D**) Interrupted sutures were used to close the tongue following debridement to remove damaged tissues.

A 54-year-old man had a dream in which a snake appeared. In Hinduism, snakes in dreams can have many connotations, so he sought the advice of an astrologer for interpretation. He was strongly advised to perform live snake worship at a nearby temple where the priest was known to house around 20 snakes of various species. For the worship to have its desired effect, the person would have to worship the same species of snake that was observed in the dream. Upon arrival at the temple, the priest discussed the dream and the snake in question was recognised as Russell’s viper based on the resemblance to a live specimen. The priest brought forth a live Russell’s viper (Figure 1A) (the identity of the snake was later confirmed by a trained herpetologist based on its morphological features) for the man to worship, instructing him to kiss the snake on the head and mimic the snake’s tongue-flickering motion. The flickering motion of the tongue and its proximity to the snake evoked a strike, resulting in the snake biting the man’s tongue on the dorsal surface. The bite immediately induced intense pain and swelling of the tongue. To tackle the effects of venom, the priest made a deep cut into the man’s tongue using his unsterile pen knife. The tongue was deeply incised but not completely removed. As a result of the intense pain, excessive bleeding and swelling, the patient was quickly brought to the emergency department at Manian Medical Centre (Erode, Tamil Nadu, India), roughly 20 min after the bite.

Upon arrival, the patient was partially conscious although unable to speak. Upon inspection, the tongue was tender to palpate, and movement was greatly restricted by the swelling and severe pain. He presented moderate respiratory distress with almost complete occlusion of the upper respiratory tract due to marked lingual and perioral oedema (Figure 1B). As typical of Russell’s viper envenomation, the patient displayed prolonged prothrombin and activated partial thromboplastin times, highlighting the perturbation of coagulation and haemostasis (Table 1). He also displayed increased levels of neutrophils and creatinine kinase. Other haematological parameters including platelet count, as well as metabolic and biochemical parameters, were within normal limits (Table 1). To counteract the envenomation effects, 100 mL (10 vials) of polyvalent antivenom raised against the Indian ‘Big Four’ venomous snakes (Bharat Serums and Vaccines, Mumbai, India) was administered intravenously (with 250 mL saline) over 60 min (at a flow rate of around 6 mL/minute). Within 10 min following admission (i.e., 30 min after the bite), the patient became visibly agitated and his oxygen saturation levels dropped, consequently losing consciousness. Airway obstruction and respiratory arrest followed. To rescue their breathing, medical staff initiated manual resuscitation with a bag valve mask, but chest expansion was not achievable. The swelling of the tongue had worsened, now protruding from the mouth. Tracheal intubation was attempted with a video laryngoscope, but the oral cavity was obstructed by the swollen tongue. The incision made to the patient’s tongue was suspected to have damaged the lingual artery at the base of the tongue resulting in profuse bleeding, inhibiting direct visualisation of the airways, and making it difficult to ascertain its anatomical orientation. Therefore, awake fibreoptic intubation with a tracheostomy as a backup was planned. Nasal intubation was achieved with a fibreoptic bronchoscope (Figure 1C) and the patient was connected to a ventilator within 45 min following the bite. The oxygen saturation levels were restored and maintained. The patient had regained consciousness and was able to follow commands.

Visual inspection of the oral cavity confirmed continuous bleeding from the incision at the posterior of the tongue. Blood and blood clots were removed from the oral cavity via a suction tube placed in the left corner of the mouth. This revealed a partial deep laceration of roughly 10 cm originating from the anterior region, traversing two-thirds the length of the tongue. Laceration of the lingual artery was confirmed as the source of the severe bleeding, and it was substantiated by the oozing of the ablated root of the tongue. The superior longitudinal muscle was completely incised with partial incision of the inferior longitudinal, transverse muscles and median fibrous septum. Following consultation with a plastic surgeon and an oral–maxillofacial surgeon, surgery was performed on the patient’s tongue. Dissection of the lacerated muscle was performed with mobilisation of the distal and proximal parts and debridement of the devitalised tissue. Following these procedures, the incised tongue was closed using simple interrupted sutures (Figure 1D). All the procedures were completed within one hour of admission. Broad-spectrum antibiotics were administered intravenously and supportive care such as physiotherapy including breath exercises and intermittent fluid suction was given. Six hours after admission, a further 100 mL (with 500 mL of saline) of polyvalent antivenom was administered over 3 h (at a flow rate of around 3.5 mL/minute) to normalise their coagulation profile (as prolonged prothrombin time was observed (Table 1)). After 24 h, the patient still displayed a prolonged coagulation profile and elevated neutrophil count, but they became normal after 72 h. Following recovery and repeated oral examinations, the patient was discharged from the hospital on the seventh day after admission. Weekly follow-ups over a period of three months identified no adverse side effects including any wounds on the tongue.

## 3. Discussion

While very few envenomings to the tongue of patients were previously reported [13,14,15,16], this is a severe case of Russell’s viper envenoming to the patient’s tongue, which resulted because of worshipping a live snake based on the advice of an astrologist. Notably, the tongue was deeply incised as a means of first aid to counteract the envenomation effects. The patient experienced the usual envenomation effects (e.g., coagulopathy) of the Russell’s viper alongside the unusual side effects of the bite being located on the tongue. As indicated above, Russell’s viper venom contains a large amount of PLA_2_ and proteolytic enzymes that act on blood coagulation [18]. As a result, they induce rapid consumption coagulopathy and subsequently, severe bleeding. In this patient, the deep incision made on the tongue following the bite exacerbated bleeding, leading to airway obstruction. The laboratory investigations indicated prolonged coagulation parameters as well as a large increase in creatinine kinase levels, suggesting significant tissue damage in the tongue, which is a skeletal muscle. Notably, the platelet level did not reduce as expected in this case due to a large amount of bleeding. While the clinicians saved this patient and their tongue, this case emphasises the urgent need to create SBE awareness among rural communities and develop/enforce appropriate policies to mitigate such extreme actions. For many people, the appearance of snakes in dreams is perceived as a bad omen. Traditional beliefs surrounding snakes go back thousands of years and are prominent in the history and mythology of almost all cultures [5,20]. Snakes are regarded as the representatives of gods, divine beings, bringers of fortune or misfortune, and their statues are worshipped in many parts of the world [4,7]. However, worshipping live venomous snakes and their mishandling is associated with serious consequences. Therefore, these aspects need attention from relevant health and forest authorities. According to the Wildlife Protection Act of 1972, it is illegal to keep snakes and other wild animals in captivity without the relevant licenses in India [21], but these rules are often overlooked in the case of religious centres [6,8]. Policy surrounding the use of venomous snakes for religious reasons requires updating to avoid hugely dangerous ceremonies such as those documented here. Therefore, the law should be enforced when necessary to mitigate such dangerous actions of using snakes for live worship and educate the communities to improve their awareness about snakes and SBE. Moreover, many misbeliefs are associated with the use of inappropriate first aid, which exacerbates SBE-induced complications [12]. In several cases, medical staff not only have to treat the complications arising from SBE but also handle the damaging effects of inappropriate first aid or treatments [22]. Indeed, incisions as well as tourniquets, blood-sucking, and burning bite sites have been proven to not be an effective first aid for SBE, although they are still being practised [9,11]. In this case, the deep incision to the tongue and laceration of the lingual artery complicated the treatment by inducing excessive bleeding and additional swelling of the tongue. This prevented the initial attempts to manually ventilate the patient. Therefore, the patient had to be nasally intubated with the help of trained healthcare professionals. Moreover, surgical procedures had to be used to reconnect the incised tongue. The clinical guidelines for restoring respiration dictate a sequential treatment plan, becoming increasingly complicated and invasive with each failed attempt [23,24]. Indeed, the difficult airway guidelines proposed by the American Society of Anaesthesiologists [25] and other authorities were not useful in this case due to severe bleeding and swelling. The available standard airway management algorithms largely depend on the visualisation of glottis directly or indirectly [26], and they were not suitable in this case due to profuse bleeding. If the medical staff were not trained to tackle this critical issue and act promptly to intubate the patient nasally then the situation would have been even worse. Similarly, the SBE treatment protocols mainly focus on tackling the envenomation effects by administering suitable antivenoms and blood products. They do not provide guidelines for managing such rare complications, which could be compounded by inappropriate first aid. Overall, this is a unique case when compared to any of the previously reported SBEs in the tongue. A snake charmer who was bitten by a Taiwan–Chinese cobra (*Naja naja atra*) on their tongue displayed minimal swelling and envenomation effects without any breathing difficulties [13]. The administration of antivenom and other supportive care successfully treated them. In contrast, a 41-year-old man who was bitten by *Crotalus atrox* displayed upper airway obstruction due to oedema of the tongue but was successfully treated with nasotracheal intubation, antivenom and supportive care [14]. A European common adder bite in a 24-year-old man caused swelling and compromised airway resulting in respiratory failure which was tackled by acute tracheotomy and antivenom treatment [16]. Sadly, a snake catcher who was bitten by a cobra in the Philippines on their tongue died promptly [27]. In a state of India, a cobra was made to bite a devotee’s tongue in a festival to please the god [8]. While this person was spitting a large amount of blood, the news article confirms that they did not suffer any further envenomation effects.

## 4. Conclusions

In conclusion, the SBE to the tongue of victims is not uncommon, although the underlying causes may vary widely. This case reports an extreme incident of a tongue bite in which the bite could have been completely avoided if the live snake worship had not taken place. Moreover, this case emphasises the extreme belief in inappropriate first aid for SBE which resulted in incising the tongue as a measure to prevent envenomation effects. Appropriate SBE awareness is critical among rural communities to mitigate the practice of live snake worship, inappropriate first aid and other ineffective traditional treatments. Moreover, healthcare professionals specifically in rural areas should be fully trained [22] and provided with appropriate guidelines to tackle such extreme cases of SBE. Importantly, healthcare professionals should be trained and educated on securing the airways using appropriate rapid management skills. The clinicians can follow the standard SBE protocols to tackle envenomation effects using appropriate antivenoms while seeking support and advice on tackling other compounding issues. Notably, the time delay should be avoided in intubating the patient with such complications as this can lead to adverse side effects. We believe that this report will improve awareness among clinicians, members of the public and relevant authorities to effectively mitigate such incidents and develop better strategies to manage these situations if they arise. We also call for a change in policy regarding the keeping of snakes in temples and other religious centres without necessary training on the safekeeping and handling of venomous snakes.

## Figures and Tables

**Table 1:** Laboratory test results for the patient at various time points. RBC, red blood cells; HCT, haematocrit; MCV, mean corpuscular volume; MCH, mean corpuscular haemoglobin; MCHC, mean corpuscular haemoglobin concentration; WBC, white blood cells; MPV, mean platelet volume; PDW, platelet distribution width; APTT, activated partial thromboplastin time; SGOT, serum glutamic oxaloacetic transaminase.

Specimen	Investigation	Results				Unit	Normal Range
		Upon Admission	After 6 Hours from Admission	After 24 Hours from Admission	After 72 Hours from Admission		
EDTA Whole Blood	Haemoglobin	15.1	-	13.2	11.0	gms%	13.0–16.0
EDTA Whole Blood	Total RBC count	4.99	-	4.32	3.76	Millions/µL	4.00–5.00
EDTA Whole Blood	HCT	42.9	-	37.7	31.3	%	41.00–50.00
EDTA Whole Blood	MCV	86.0	-	87.3	83.2	fl	81.10–96.00
EDTA Whole Blood	MCH	30.3	-	30.6	29.3	pg	27.20–33.20
EDTA Whole Blood	MCHC	35.2	-	35.0	35.1	%	32–36
EDTA Whole Blood	Total WBC count	12.81	-	13.61	6.91	×10^3^ Cells/µL	4.00–11.00
EDTA Whole Blood	Neutrophils	11.49	-	14.98	4.04	×10^3^ Cells/µL	2.0 to 7.0
EDTA Whole Blood	Lymphocytes	0.86	-	0.82	1.81	×10^3^ Cells/µL	1.0 to 3.0
EDTA Whole Blood	Monocytes	0.39	-	0.76	0.53	×10^3^ Cells/µL	0.1 to 0.8
EDTA Whole Blood	Eosinophils	0.03	-	0.00	0.46	×10^3^ Cells/µL	0.02 to 0.5
EDTA Whole Blood	Basophils	0.04	-	0.05	0.07	×10^3^ Cells/µL	0.02 to 0.1
EDTA Whole Blood	Neutrophils	89.8	-	90.2	58.4	%	55–75
EDTA Whole Blood	Lymphocytes	6.7	-	4.9	26.2	%	15–30
EDTA Whole Blood	Eosinophils	0.2	-	0.0	6.7	%	1–5
EDTA Whole Blood	Monocytes	3.0	-	4.6	7.7	%	2–10
EDTA Whole Blood	Basophils	0.3	-	0.3	1.0	%	Up to 1
EDTA Whole Blood	Platelet Count	276	-	173	181	×10^3^ Cells/µL	150–450
EDTA Whole Blood	MPV	12.4	-	12.9	11.8	fl	6.5–12.0
EDTA Whole Blood	PDW	16.8	-	14.0	15.3	fl	9.0–13.0
Serum	Urea	31.0	-	30.0	-	mg/dL	15–40
Serum	Creatinine	0.88	-	1.12	-	mg/dL	0.7–1.4
Serum	Uric Acid	6.1	-	5.6	-	mg/dL	3.4–7.2
Serum	Creatinine kinase	670	-	-	-	U/L	24–195
Serum	Bilirubin (total)	1.27	-	-	0.63	mg/dL	0.2–1.2
Serum	Bilirubin (direct)	0.82	-	-	0.18	mg/dL	0–0.2
Serum	Bilirubin (indirect)	0.45	-	-	0.45	mg/dL	0.2–0.9
Serum	SGOT	30	-	-	17	U/L	5–35
Citrated plasma	Prothrombin time	Prolonged	23.1	26.0	14.4	Seconds	11.5–16.0
Citrated plasma	APTT	Prolonged	35.0	44.0	31.7	Seconds	26.0–40.0

## Data Availability

Not applicable—as all data from this study are included within this article.
